# Physicochemical characteristics and antioxidant stability of spray‐dried soy peptide fractions

**DOI:** 10.1002/fsn3.3381

**Published:** 2023-04-21

**Authors:** Zahra Akbarbaglu, Fardin Tamjidi, Khashayar Sarabandi, Ali Ayaseh

**Affiliations:** ^1^ Department of Food Science, College of Agriculture University of Tabriz Tabriz Iran; ^2^ Department of Food Science & Engineering, Faculty of Agriculture University of Kurdistan Sanandaj Iran; ^3^ Department of Food Science & Technology, School of Medicine Zahedan University of Medical Sciences Zahedan Iran

**Keywords:** antioxidant stability, microencapsulation, morphological changes, soybean peptide fractions, spray‐drying

## Abstract

The direct addition of health‐promoting peptides to food products is limited due to their physicochemical instability and bitter taste as well as their bio‐functionality may be influenced by M_*W*_. In this study, SPI hydrolysate (SPIH) was Alcalase‐prepared, size‐fractionated (<10, 10–30, and 30–100 kD), and the amino acid composition of peptide fractions determined. The physicochemical properties, morphology, and antioxidant stability of the fractions were also investigated after spray‐drying encapsulation in maltodextrin‐WPC carrier. The two low M_*W*_ peptide fractions (especially, PF < 10) were more active than intact SPI, SPIH, and high M_*W*_ peptide fraction in scavenging free radicals and chelating transition metal ions. As compared to the particles containing SPIH, those containing the smallest peptide fraction (PF < 10) had higher solubility and hygroscopicity, lower production yield and wettability, and more wrinkles, indentations and surface roughness. The highest antioxidant stability during spray‐drying was observed for the two low M_*W*_ peptide fractions, which examined by scavenging of free radicals of DPPH (88%), ABTS (97%), OH (93%) and NO (80%), chelating of iron (88%) and copper (87–90%) ions, reducing power (93%), and total antioxidant activity (90%). This finding reflects more structural and biological stability of the low M_*W*_ fractions to shear stress and dehydration during spray‐drying, as compared with SPIH. The spray‐drying encapsulated soy peptide fractions may be used as nutraceuticals for the development of functional foods.

## Graphical abstract

The Alcalase hydrolysate of soy protein was size‐fractionated (<10, 10–30, and 30–100 kD) and spray‐drying encapsulated. The production yield and characteristics of spray‐dried peptides influenced by their molecular weight. The antioxidant stability of spray‐dried peptides increased with decreasing their molecular weight.

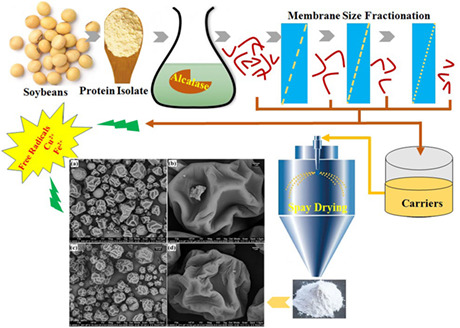

## 1 INTRODUCTION

Nowadays, with increasing human population and industrialization, and changing lifestyle, there are some concerns regarding to poor quality of foods and prevalence of chronic diseases. Therefore, scientists and manufacturers are trying to produce functional foods by fortifying food products with natural bioactive compounds. Some of the most common health‐promoting substances include polyphenols, carotenoids, anthocyanins, vitamins, minerals, probiotics, fatty acids, phytosterols, and bioactive peptides.

Proteins are inexpensive, economic, available, and rich sources of bioactive peptides. Protein‐derived bioactive peptides are specific encrypted and inactive fragments within the parent protein, which may exert various health‐enhancing functions after release by different methods, such as enzymolysis (Udenigwe, 2014). Some of their beneficial physiological effects include anti‐aging, anti‐cancer, anti‐cariogenic, antidiabetic, anti‐hypertensive, anti‐microbial, anti‐oxidative stress, anti‐inflammatory, cholesterol lowering, growth enhancing, immunomodulatory, mineral binding, radical scavenging, and regulation of glucose‐insulin homeostasis and satiety (Wang & Selomulya, 2020). The fortification of foods with peptides is also valuable for populations with special nutritional needs (e.g., infants, athletes, and the elderly) owing to their ease of digestion (Michaelidou, 2008). Moreover, some peptides may have superior techno‐functional characteristics. Nevertheless, several challenges, such as high hygroscopicity, physicochemical instability, perishability, and cross‐reactivity with other compounds, limit the direct use of peptides in food and dietary supplement formulations. Another disadvantage of peptides is their unpleasant bitter taste, which reduces the sensory acceptability of fortified products (Mohan et al., 2015; Udenigwe, 2014). Therefore, it is necessary to use techniques to minimize these challenges, and microencapsulation is the most important technology for this purpose.

The microencapsulation or delivery technology itself includes several methods, of which spray‐drying is the most common and economical, and is commercially available on a large scale. Spray‐drying converts various water‐based feeds containing bioactive compounds (e.g., extracts, solutions, and dispersions) into flowable powders (Jafari et al., 2021), and fixes the core materials within a particulate carrier matrix in solid state. Therefore, considerable research efforts have been conducted on the spray‐drying microencapsulation of bioactive peptides and protein hydrolysates. However, the complex structure and specific physicochemical characteristics of peptides/proteins make them sensitive to the environmental stresses encountered during spray‐drying. For example, the mechanical stresses created by atomization and by binding these surface‐active components to air–liquid interface, as well as the thermal and dehydration stresses during spray‐drying, lead to conformational changes, denaturation, aggregation, and thereby inactivation of them (Ajmera & Scherließ, 2014). That is why different strategies, such as modifying the composition of feed formulation and using various carriers (e.g., proteins, polysaccharides, sugars, amino acids, and surfactants), have been applied to protect peptides during spray‐drying (Mohan et al., 2015).

Soybeans as an excellent and inexpensive source for protein and health‐promoting compounds have a high world annual production (more than 353 million metric tons in 2020; FAOSTAT, 2023) and a special value in diets. Soybean peptides have important functional characteristics, such as good solubility, emulsifying ability, and biological activity (Liu et al., 2012). It has been reported that spray‐drying is more effective than freeze‐drying for microencapsulation of non‐fractionated soybean hydrolysates in soy protein isolate–maltodextrin mixture, and yields a final product of better quality (e.g., solubility, flowability, and bitterness‐masking) (Wang et al., 2020).

The biological activity and techno‐functional characteristics of peptides show a complex dependence upon their molecular weight (M_*W*_) distribution, amino acid composition and sequence, degree of hydrophobicity, and solubility (Phongthai et al., 2018). The M_*W*_ of the peptides to be spray‐dried can influence on the quality characteristics of final powder due to having different surface activity and behavior at air–liquid interface during drying. Since there is sparse information about this issue in literature, this study aimed to investigate the amino acid composition, antioxidant capacity, and spray‐drying encapsulation of size‐fractionated soy protein hydrolysates, and to assess the remaining antioxidant activity, and physicochemical and morphological characteristics of the spray‐dried peptides.

## 2 MATERIALS AND METHODS

### 2.1 Materials

Alcalase® 2.4 L, soy protein isolate (SPI), and maltodextrin (DE18‐20) were purchased from Novo (Nordisk, Bagsvaerd, Denmark), Jun Kai Chemical Co. (Zhengzhou, Henan Province, China), and Pooran Powder Sepahan Co. (Isfahan, Iran), respectively. WPC (80% protein) was obtained from Arla Co. (Denmark). Sodium nitroprusside, ferrozine 3‐(2‐pyridyl)‐5‐6‐diphenyl‐1,2,4‐triazine‐4′,4″ disulfonic acid sodium salt, pyrocatechol violet, naphthyl‐ethylene‐diamine‐dihydrochloride (NEDD), sulfanilamide, 2,2′‐azino‐bis(3‐ethylbenzothiazoline‐6‐sulfonic acid) diammonium salt (ABTS), and 1,1‐diphenyl‐2‐picrylhydrazyl (DPPH) were from Sigma–Aldrich Co. (St. Louis, MO, USA). Alpha‐deoxyribose was supplied from Fluka (Stockholm, Sweden), and all other chemicals used were from Merck Co. (Darmstadt, Germany).

### 2.2 Enzymolysis of SPI

SPI (5% w/v) was dissolved in phosphate buffer solution (PBS, 0.01 M, pH 8) under magnetic stirring at 50°C for 30 min. Then, Alcalase (2% v/v) was added to it, and stirred at 50°C and pH 8, for 120 min. Afterward, the enzyme was inactivated by immersing the reaction flask in a hot water bath (95°C) for 15 min. Finally, the undigested proteins were precipitated by centrifugation (7000 g, 10 min), and the supernatant containing SPI hydrolysate saved (Akbarbaglu et al., 2022).

### 2.3 Fractionation of SPI hydrolysates

A portion of the supernatant was passed through ultrafiltration membrane filters (Amicon® Ultra centrifugal filter, Millipore, UK) with M_*W*_ cutoff of 100 kD, 30 kD, and 10 kD, to obtain peptide fractions of PF‐30–100 (30–100 kD), PF‐10–30 (10–30 kD), and PF < 10 (<10 kD), respectively. The resulting peptide fractions, along with another portion of supernatant (i.e., SPI hydrolysate: SPIH), were freeze‐dried (Christ, Germany; −20°C, 0.1 mbar) and preserved at −18°C for further use.

### 2.4 Amino acid composition

SPI, SPIH, and peptide fractions were hydrolyzed in 6 N HCl at 110°C for 24 h, then their amino acid composition assessed by an HPLC system equipped with reversed‐phased column (Novapack C18, 4 μm, Waters, Milford, MA) and reported as mg/g dry sample (You et al., 2010). The tryptophan content of samples was determined after alkaline hydrolysis.

### 2.5 Spray‐drying encapsulation of peptide fractions

The SPI peptide fractions were microencapsulated by spray‐drying technique as following. A 2 g portion of each freeze‐dried peptide fraction was dissolved in 20 mL PBS (0.1 M, pH = 7.4) and mixed with 80 mL of carrier solution (20% w/v of maltodextrin and WPC in equal ratio). The final solution was then microencapsulated by feeding into a lab‐scale spray dryer (Büchi B‐191; Büchi Laboratoriums‐Tecnik, Switzerland) under the following process parameters (Sarabandi et al., 2022): inlet‐air temperature (130 ± 1°C), outlet‐air temperature (82 ± 2°C), feed rate (5 mL/min), drying air flow rate (0.54 m^3^ h^−1^), nozzle diameter (0.7 mm), and air pressure (5.6 bar). The spray‐dried powders were packed in airtight bags and kept in refrigerator until use.

The powder production yield (% w/w) during spray‐drying was calculated by dividing the weight of the obtained powder by the total weight of the primary solid matter and then multiplying by 100.

### 2.6 Antioxidant characterization

The activity of SPI and freeze‐dried SPIH and peptide fractions in scavenging radicals of DPPH, ABTS, hydroxyl and nitric oxide, and chelating prooxidant transition metal ions of iron and copper, their reducing power, and their total antioxidant activity (TAA) were measured in the same manners as described by Sarabandi and Jafari (2020). The retention percentage (%*R*) of each of the above‐mentioned antioxidant activity indices during spray‐drying was calculated from equation: %*R* = (*A*_2_/*A*_1_) × 100, where A_1_ and A_2_ are the values of that index in the spray dryer feed and in the corresponding reconstituted powder, respectively.

### 2.7 Physicochemical properties of spray‐dried powders

The moisture content, water activity (A_*W*_), bulk and tapped densities, solubility, wettability, and hygroscopicity of spray‐dried powders were measured according to the methods of Vonghirundecha et al. (2022), and their flowability indices (angle of repose, Hausner ratio, and Carr's index) were examined according the methods of Sarabandi and Jafari (2020).

### 2.8 Morphology of spray‐dried powders

The morphology of spray‐dried powders was assessed using a Hitachi PS‐230 scanning electron microscope (SEM) under 25 kV accelerating voltage after coating with a gold film.

### 2.9 Statistical analysis

The experiments were performed in triplicate, and the data were reported as mean ± SD and analyzed by one‐way ANOVA using SPSS software ver. 19.0 (SPSS Inc., Chicago, IL). The Duncan's test was employed to determine statistical differences (*p* < .05) between selected treatments.

## 3 RESULTS AND DISCUSSION

### 3.1 Amino acid composition

The amino acid composition is a key factor influencing biological activity and functional characteristics of peptides. Table 1 shows the amino acid composition of intact‐SPI, SPIH, and SPI peptide fractions with different M_*W*_ distribution (PF < 10, PF‐10–30, PF‐30–100). No obvious difference was observed in amino acid composition of these samples. Considering that Alcalase is a nonspecific protease (Yu & Mikiashvili, 2020), it is the difference in M_*W*_ of the hydrolysates fractions that mainly affects their characteristics in this study. Glutamic acid, aspartic acid, arginine, leucine, and lysine were the predominant residues in SPI and its hydrolysates, and their total hydrophobic and antioxidant amino acids (HAA and AAA) were found to be about 34% and 16%, respectively. In another study, different endopeptidases (Alcalase, Flavourzyme, Thermolysin Proteinase K, and Pepsin + Pancreatin) were employed to hydrolyze rapeseed protein isolate, and it was found that Alcalase produces the highest yield of protein hydrolysate and its hydrolysate contains significant levels of HAA (28%) and AAA (6.7%) (He et al., 2013).

**TABLE 1:** Amino acid composition of SPI, SPIH, and peptide fractions (mg amino acid/g dry sample).

Amino acid	SPI	SPIH	PF < 10	PF‐10–30	PF‐30–100
Aspartic acid	97.6	97.3	97.1	97.5	97.9
Glutamic acid	153.1	153.2	152.7	153.0	152.8
Histidine	19.7	19.8	19.9	19.8	19.9
Serine	43.2	42.5	42.6	42.7	43.1
Arginine	53.4	53.7	53.9	53.7	53.8
Glycine	35.7	35.3	34.7	34.9	35.5
Threonine	30.2	29.7	30.0	30.6	30.5
Alanine	36.3	36.5	36.4	36.3	36.4
Tyrosine	27.9	28.1	28.5	28.4	28.2
Methionine	10.6	10.7	10.7	10.8	10.4
Valine	32.8	32.5	32.8	32.4	32.6
Phenylalanine	39.5	39.6	40.1	39.9	40.0
Isoleucine	30.2	30.4	30.5	30.3	30.6
Leucine	55.1	55.0	55.3	55.2	55.1
Lysine	48.3	48.9	49.1	48.8	48.5
Tryptophan	10.9	11.1	11.0	10.7	10.9
HAA^a^	243.3	243.9	245.3	244.0	244.2
AAA^b^	117.4	118.6	119.2	118.5	117.9
TAA^c^	724.5	724.3	725.3	725.0	726.2

^a^ HAA: Hydrophobic amino acids = alanine, valine, isoleucine, leucine, tyrosine, phenylalanine, tryptophan, and methionine;

^b^ AAA: Antioxidant amino acids = tryptophan, methionine, histidine, tyrosine, and lysine;

^c^ TAA: Total amino acids.

### 3.2 Antioxidant activity

#### 3.2.1 DPPH and ABTS radicals scavenging

**FIGURE 1:**
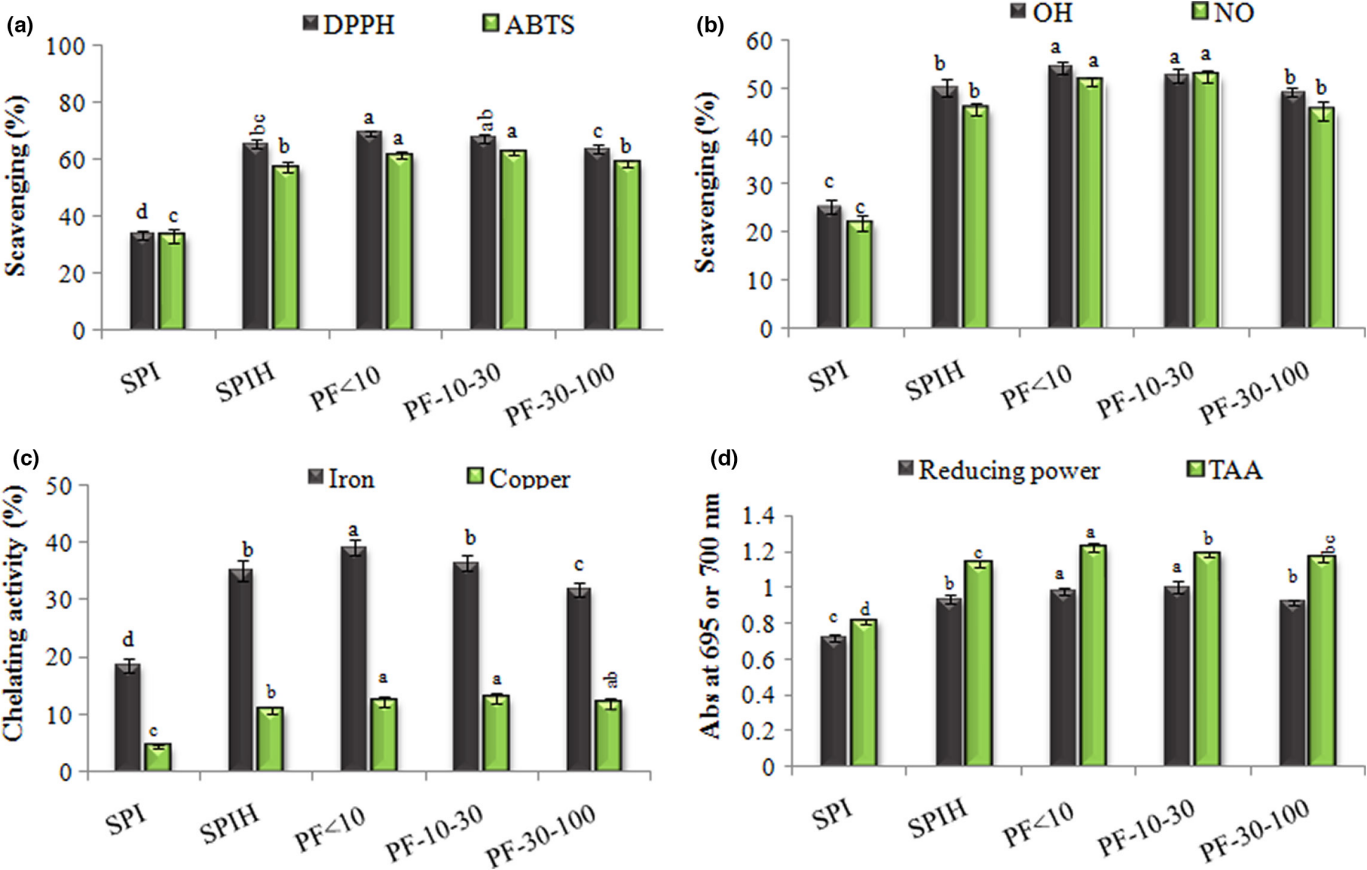
The antioxidant activity of SPI, SPIH, and soy peptide fractions (PF < 10, PF‐10–30, and PF‐30–100), in terms of scavenging of DPPH, ABTS (a), OH and NO (b) radicals, chelating of Fe and Cu ions (c), reducing power and total antioxidant activity (TAA; d).

The capacity of intact‐SPI, unfractionated hydrolysate (i.e., SPIH), and peptide fractions with different M_*W*_ distribution in inhibiting DPPH and ABTS free radicals were examined (Figure 1a). The SPIH was significantly more efficient than intact‐SPI in scavenging free radicals of DPPH (65% vs. 33%) and ABTS (57% vs. 32%). Also, the peptide fractions PF < 10 and PF‐10–30 had more DPPH and ABTS scavenging activities than fraction PF‐30–100 (Figure 1). The M_*W*_ of peptides plays a decisive role in their antioxidant activity (Islam et al., 2021), because the free amino acid content, structure flexibility, and accessibility of reactive side chains increase with decreasing M_*W*_ of hydrolysate. Moreover, the presence of special free amino acids with ability to donate electrons (like hydrophobic and antioxidant ones) and thereby to convert DPPH free radicals into stable diamagnetic molecule can be another reason (Xie et al., 2019). For the same reasons, the peptide fractions with low M_*W*_ had high capacities for inhibiting ABTS radicals (Moghadam et al., 2020). In a similar research work, the enzymatic hydrolysate of cod protein was size‐fractionated, and it was found that the low M_*W*_ fraction (<3 kD) has higher levels of free and antioxidant amino acids as compared to intact protein, unfractionated hydrolysate, and the high M_*W*_ fraction (5 < kD) (Farvin et al., 2016). In another study, the Flavourzyme‐hydrolyzed spent hen meat was size‐fractionated, and it was found that the smallest peptide fraction (<5 kD) has the highest antioxidant activity (Kumar et al., 2022).

#### 3.2.2 HO^·^ and NO^·^ radicals scavenging

The reactive oxygen species (ROS) of hydroxyl and nitric oxide radicals have damaging effects on the vital biomolecules, it is therefore very important to quench them in biological systems. The capacities of intact‐SPI, SPIH, and peptide fractions with different M_*W*_ distribution in scavenging HO^·^ and NO^·^ free radicals were investigated (Figure 1b).

Enzymatic hydrolysis increased SPI capacity to scavenge HO^·^ radicals (from ~25 to ~50%). Moreover, fractions PF < 10 and PF‐10–30 had higher HO^·^‐inhibitory activity than SPIH and fraction PF‐30–100 (Figure 1b). Factors such as the high degree of hydrolysis (i.e., emerging low M_*W*_ peptides) and the release of electron‐donor free aromatic amino acids (e.g., tryptophan, tyrosine, phenylalanine) are involved in inhibition of hydroxyl radicals (Xie et al., 2019). Similar results have also been reported regarding to the HO^·^‐inhibitory activity of calabash nutmeg (Akinyede et al., 2021) and flaxseed (Sarabandi & Jafari, 2020) proteins after enzymolysis and hydrolysate fractionation.

Excessive levels of NO^·^ free radicals lead to the production of pro‐inflammatory cytokines and occurrence of several diseases such as atherosclerosis, chronic inflammation, rheumatoid arthritis, Parkinson's disease, and cancer (Ahn et al., 2012). In this research, the enzymatic hydrolysis of SPI significantly increased its ability in the inhibition of NO^·^ radicals (from ~22 to ~45%). Moreover, fractions PF < 10 and PF‐10–30 showed the highest NO^·^‐inhibitory activity (~52%) (Figure 1b). This can be attributed to the release of hydrophobic (e.g., leucine, isoleucine, alanine, phenylalanine, and valine) and antioxidant amino acids (Akbarbaglu et al., 2022), and/or exposing of their functional groups after enzymatic hydrolysis. Similar results have also been reported for enzymatically hydrolyzed proteins of black bean (López‐Barrios et al., 2016) and legume (Xu et al., 2021).

#### 3.2.3 Iron and copper ions chelating

The use of natural chelating agents in food formulations can have a great influence on preventing the destructive reactions accelerated/mediated with metal ions, especially lipid oxidation. In this research, enzymolysis of SPI and fractionation of the hydrolysate had a significant effect on the activity of peptide materials in chelating iron and copper ions (Figure 1c). The iron chelation percentage by SPIH and fractionated peptides was much higher than that by intact‐SPI, and fraction PF < 10 was most effective in this regard (*p* < .05). In the same way, the copper chelating percentage by SPIH and peptide fractions was much higher than that by the original protein, but the peptide fractions had the same effectiveness. The higher chelating activity of SPIH and peptide fractions can be attributed to the release of acidic and basic amino acids containing an extra carboxylic or amino group (Shahidi & Zhong, 2015) together with the release of more of these groups by hydrolysis of peptide bonds, which can coordinate or form complexes with iron and copper ions through their lone pair electrons (Lindsay, 2017). In similar studies, it has been observed that the metal ion chelating activity of proteins of grass turtle (Islam et al., 2021), rice bran (Phongthai et al., 2018), and calabash nutmeg (Akinyede et al., 2021) is influenced by enzymatic hydrolysis, amino acid composition, and M_*W*_ of peptide fractions.

#### 3.2.4 Reducing power and TAA

The reducing power and TAA of SPI, SPIH, and peptide fractions were also investigated (Figure 1d). The reducing power of the samples significantly increased in the order of intact‐SPI*―*SPIH, PF‐30–100*―*PF‐10–30, and PF < 10. Enzymatic hydrolysis plays a significant role in antioxidant activity of proteins by decreasing M_*W*_ of polypeptides and increasing free amino acids number (e.g., histidine, tyrosine, methionine, tryptophan, and lysine). The presence of a large number of electron‐donor or antioxidant amino acid groups in fractions with lower M_*W*_ can be considered as a reason for their higher reactivity (Islam et al., 2021). Similar results have been reported for the enzymatic hydrolysates and peptide fractions obtained from calabash nutmeg (Akinyede et al., 2021) and flaxseed (Sarabandi & Jafari, 2020) proteins.

The TAA of samples was evaluated by measuring the reaction absorption due to the formation of the green phosphomolybdenum complex in acidic conditions (Figure 1d). The findings showed that enzymatic hydrolysis of protein had a significant effect on TAA. The TAA was found to be 0.81, 1.13, and 1.23 for intact‐SPI, SPIH, and fraction PF < 10, respectively. An increase in TAA has also been reported for proteins of flaxseed (Sarabandi & Jafari, 2020) and black bean (Aguilar et al., 2019) after enzymatic hydrolysis with different proteases.

### 3.3 Characterization of spray‐dried peptides

The effect of core material type (i.e., SPIH and peptide fractions with different M_*W*_ distribution) on some physical, techno‐functional, and stability characteristics/indices of the spray‐dried powders was investigated (Tables 2 and 3).

**TABLE 2:** Physicochemical and functional properties of spray‐dried SPIH and peptide fractions.

Sample	Yield (%)	Moisture (%)	Aw	Solubility (%)	Wettability (sec)	Hygroscopicity (%)
SPIH	73.80 ± 3.75^a^	2.77 ± 0.35^b^	0.190 ± 0.01^b^	90.67 ± 2.45^b^	14.90 ± 3.05^a^	25.27 ± 2.51^b^
PF < 10	58.93 ± 4.07^c^	3.53 ± 0.30^a^	0.260 ± 0.01^a^	95.10 ± 1.77^a^	9.77 ± 1.45^b^	30.33 ± 1.90^a^
PF‐10–30	65.63 ± 2.31^b^	3.67 ± 0.25^a^	0.260 ± 0.02^a^	93.00 ± 1.25^ab^	11.50 ± 0.90^ab^	28.00 ± 1.61^ab^
PF‐30–100	68.23 ± 2.51^ab^	3.27 ± 0.15^ab^	0.257 ± 0.02^a^	91.60 ± 0.92^b^	10.93 ± 2.58^ab^	25.20 ± 1.31^b^

*Note*: Data are presented as mean ± SD (*n* = 3), and values denoted by different letters within each column are significantly different (*p* < .05).

**TABLE 3:** Flowability properties of spray‐dried SPIH and peptide fractions.

Sample	Bulk density (g/mL)	Tapped density (g/mL)	Angle of repose (°)	Hausner ratio	Carr's index
SPIH	0.553 ± 0.01^a^	0.667 ± 0.01^a^	32.67 ± 1.53^b^	1.203 ± 0.01^b^	0.170 ± 0.01^b^
PF < 10	0.500 ± 0.01^b^	0.627 ± 0.01^b^	36.67 ± 1.53^a^	1.250 ± 0.01^a^	0.200 ± 0.01^a^
PF‐10–30	0.510 ± 0.01^b^	0.637 ± 0.01^b^	36.00 ± 2.00^a^	1.247 ± 0.01^a^	0.200 ± 0.01^a^
PF‐30–100	0.520 ± 0.01^b^	0.640 ± 0.01^b^	34.67 ± 1.53^ab^	1.230 ± 0.01^ab^	0.187 ± 0.01^ab^

*Note*: Data are presented as mean ± SD (*n* = 3), and values denoted by different letters within each column are significantly different (*p* < .05).

Powder production yield is a measure of process economic efficiency; its maximum (~74%) and minimum (~59%) levels were observed for the feeds containing SPIH and fraction PF < 10, respectively (Table 2). This finding reflects that small peptides increase the particle adhesion during spray‐drying, probably due to their reduced glass transition temperature (Zhou et al., 2014). The moisture content and water activity (Aw), as measures of physicochemical and microbial stability, were fallen in the ranges of 2.8–3.7% and 0.19–0.26 for the powders, respectively. These values imply the microbial stability of spray‐dried peptides (Ortiz et al., 2009).

The solubility of the spray‐dried peptide fractions improved by decreasing their M_*W*_. This finding implies the effect of M_*W*_ of peptides on the accessibility of their hydrophilic regions and thereby on their water adsorption and solubility (Islam et al., 2021; Moghadam et al., 2020). However, the peptide fractionation had no significant effect on wettability of the spray‐dried fractions. The hygroscopicity of the spray‐dried powders was influenced by M_*W*_ of the peptide fraction encapsulated in them (Table 2). In agreement with the solubility data, the hygroscopicity increased with decreasing M_*W*_ of peptides. This finding can be a result of a more increase in the exposed net charge density in low M_*W*_ fractions (Netto et al., 1998) and in the number of their low M_*W*_ components which may affect the water sorption pattern through colligative effects (Zhou et al., 2014). The bulk density, tapped density, angle of repose, Hausner ratio, and compressibility index (Carr's index) of the spray‐dried powders were found in the ranges of 0.55–0.55 g/mL, 0.62–0.67 g/mL, 32–37°, 1.21–1.25, and 0.17–0.2 respectively, and M_*W*_ of the encapsulated peptide fraction had no significant effect on these values (Table 3). Anyway, these data indicate a proper flowability for the powders particles (Akbarbaglu et al., 2019).

In another study, the hydrolysates of stripped weakfish were microencapsulated in maltodextrin by spray‐drying, and it was found that the type of hydrolysate (with Alcalase or Protamex) influences the production efficiency (73–76%), moisture content (5.3–6.1%), and Aw (0.11–0.13) of the powders, and the hygroscopicity of the hydrolysates decreases from 30 to 16.1–17.1%, after encapsulation (Lima et al., 2021). It has been reported that the physicochemical, functional, and flowability characteristics of encapsulated hydrolysate powders are influenced by the composition of formulation (especially, type and concentration of hydrolysate and carrier) and the microencapsulation technique (e.g., spray‐ or freeze‐drying) (Akbarbaglu et al., 2019; Gan et al., 2020; Kleekayai et al., 2022; Sarabandi & Jafari, 2020).

### 3.4 Morphological characterization

**FIGURE 2:**
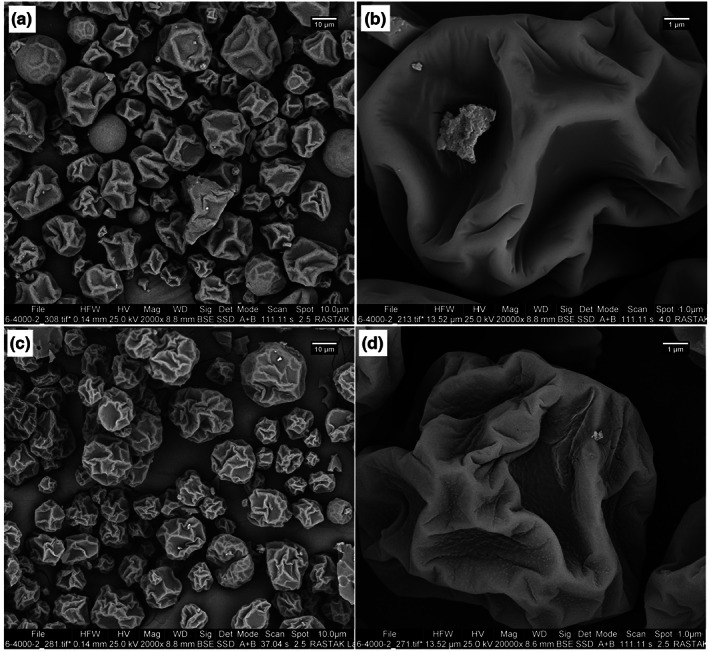
SEM images of microparticles containing SPIH (a, b) or the smallest soy peptide fraction (PF < 10; c, d).

The effect of core material type (SPIH or PF < 10) on the morphological characteristics of spray‐dried powder particles was investigated (Figure 2). The SEM images indicated the presence of relatively uniform‐sized particles with wrinkled and irregular structures in both powder samples. The SPIH‐containing particles had smooth surfaces despite having indented structures, but those containing PF < 10 had rough surfaces and more wrinkles and indentations. Aggregated spherical structures with rough surfaces, and irregular structures with rough and wrinkled surfaces have, respectively, been observed for encapsulated hydrolysates of chicken‐breast protein (Setthaya et al., 2021) and stripped weakfish (Lima et al., 2021), after spray‐drying with maltodextrin carrier.

Yielding particles with different sizes and shapes is a characteristic of spray‐drying technique (Jafari et al., 2021). But the difference in surface shrinkage and roughness of particles can be attributed to the higher mobility of peptides with low M_*W*_ (PF < 10), which allows them to rapidly migrate to the interface and to the outer layer of droplets during dehydration (Ajmera & Scherließ, 2014). The difference in emulsifying properties of peptides and intact protein is also evident during the production and stabilization of emulsions (Islam et al., 2021). However, some researchers attributed the rough surface of spray‐dried powders to slow film formation during the drying and cooling phases of the process (Kurozawa et al., 2009).

### 3.5 Retention of antioxidant activity in spray‐dried peptides

**FIGURE 3:**
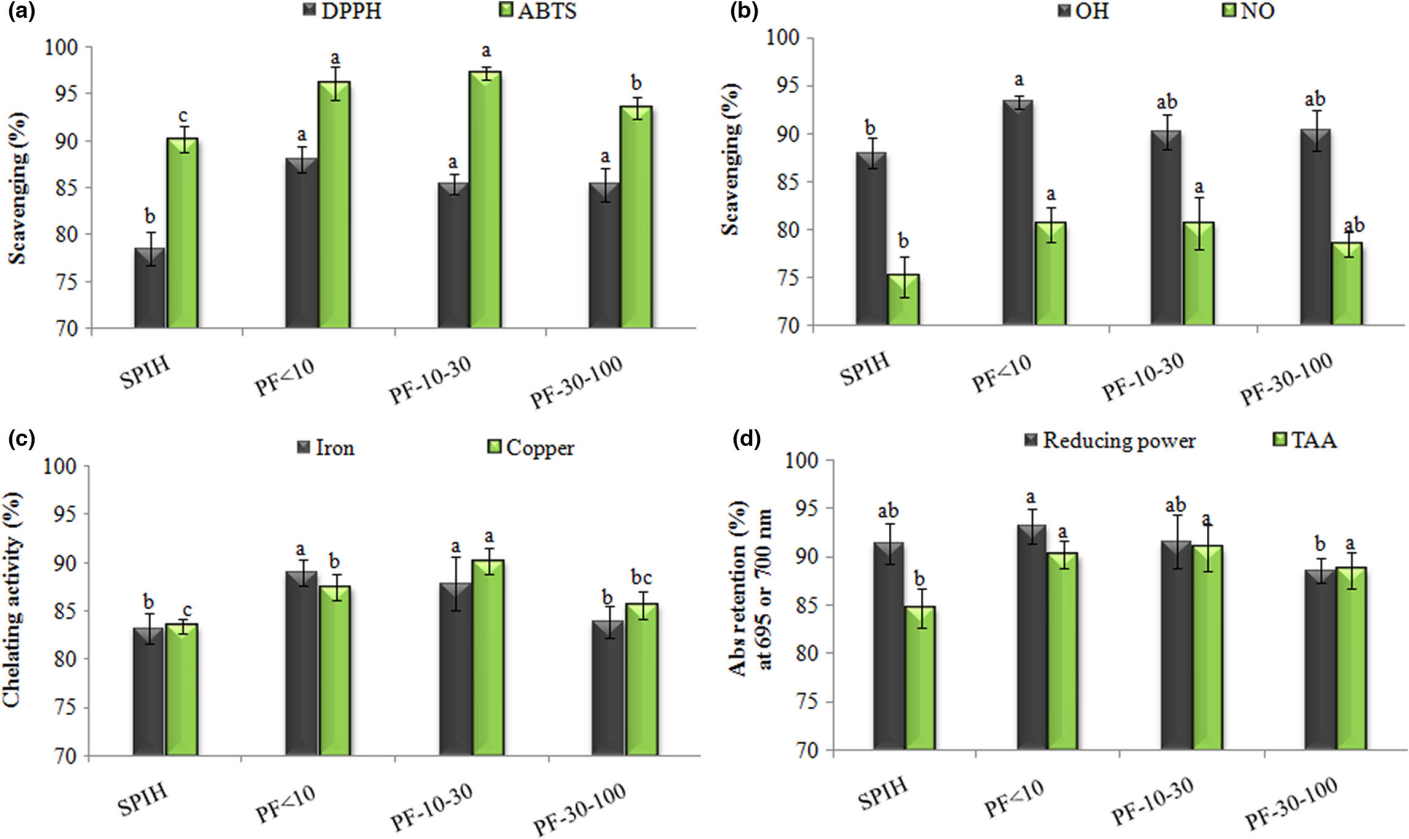
The retention percentage of antioxidant activity in SPI, SPIH, and soy peptide fractions (PF < 10, PF‐10–30, and PF‐30–100) after spray‐drying encapsulation, in terms of scavenging of DPPH, ABTS (a), OH and NO (b) radicals, chelating of Fe and Cu ions (c), reducing power and total antioxidant activity (TAA; d).

One of the most important goals of microencapsulation is to stabilize and preserve the biological activity of bioactive substances. In this study, the antioxidant activity retention of the peptide samples (SPIH, PF < 10, PF‐10–30 and PF‐30–100) after spray‐drying encapsulation was investigated. Figure 3 shows the retention percentage of antioxidant activity in the spray‐dried peptides. With all antioxidant activity assays, 75–97% of initial antioxidant activity of free peptides preserved after spray‐drying, and the fractionated peptides generally had more antioxidant stability than SPIH. Regardless of the type of core material, the highest and lowest antioxidant stability was related to ABTS (97%) and NO^·^ (75%) radical scavenging, respectively. The microcapsule powders of PF < 10 and PF‐10–30 had the same antioxidant stability, except in copper ion chelating assay; their antioxidant stability was also higher than that of PF‐30–100 powder in terms of ABTS radical scavenging, transition metal ion chelating and reducing power. The decrease in antioxidant activity of peptides during spray‐drying can be attributed to the destruction (Akbarbaglu et al., 2019) or complexation/blocking of some of their antioxidant active sites. A reason for the high stability of low M_*W*_ peptides is that, unlike those with high M_*W*_ in SPIH and PF‐30–100 which undergo conformational changes, they are resistant to the shear and dehydration stresses during spray‐drying (Ajmera & Scherließ, 2014). The difference in amino acid sequence and heat sensibility of peptides can be another reason. That's why, the retention percentages of immune‐related proteins (like lactoferrin) in caprine and camel milk (15–5%) (Zhang et al., 2016) and the antioxidant stability in hydrolysates of casein (76–95%) (Sarabandi et al., 2018) and flaxseed protein (62–91%) (Sarabandi & Jafari, 2020) have been different after spray‐drying. The drying method type (oven‐, spray‐, or freeze‐drying) have also had a significant effect on the retention of antioxidant/biological activity in the hydrolysates of *Perinereis aibuhitensis* (Liu et al., 2022) and edible bird's nest (Gan et al., 2020).

## 4 CONCLUSION

The direct fortification of food formulations and diets with protein hydrolysates is limited due to their physicochemical instability and bitter taste, as well as, their bioactivity may be influenced by M_*W*_. In this research, SPI hydrolysates were prepared by alcalase, then size‐fractionated and microencapsulated in maltodextrin‐WPC carrier by spray‐drying. The physical, techno‐functional, and stability characteristics/indices of the spray‐dried powders were influenced by the M_*W*_ of encapsulated peptides. As comparing to the SPIH microcapsules, those containing low M_*W*_ peptides (<10 kD) had higher indentation, wrinkling, and surface roughness; this may be due to the difference in their solubility and surface activity. The low M_*W*_ peptide fractions (especially, PF < 10) generally had the highest capacity in scavenging free radicals and chelating transition metal ions, and the highest antioxidant activity retention after spray‐drying; the high M_*W*_ peptide fraction (i.e., PF‐30–100) and the unfractionated hydrolysates may have undergone structural and biological changes during spray‐drying. Fractionation and encapsulation of protein hydrolysates may be important to select more effective peptides and to improve their utilization, as lower concentration of them will be required for the development of functional foods and thereby little change seen in the finished products.

## AUTHOR CONTRIBUTIONS

**Zahra Akbarbaglu:** Formal analysis (lead); investigation (lead); validation (lead); writing – original draft (lead). **Fardin Tamjidi:** Conceptualization (lead); funding acquisition (lead); resources (equal); supervision (lead); visualization (lead); writing – review and editing (equal). **Khashayar Sarabandi:** Data curation (lead); project administration (lead); writing – review and editing (equal). **Ali Ayaseh:** Methodology (lead); project administration (supporting); resources (equal).

## Data Availability

All data generated or analyzed in this article are included within it.
